# Use of intraoperative neuromonitoring in robotic thyroidectomy: a systematic review of recurrent laryngeal nerve outcomes

**DOI:** 10.1097/MS9.0000000000004717

**Published:** 2026-01-22

**Authors:** Nagham Al Dirani, Hadi Ftouni, André Chemaly, Abbass Al Bazzal, Fatima Youness, Jana Kotaich, Linda AbouAbbas

**Affiliations:** a Faculty of Medical Sciences, Lebanese University, Hadath, Lebanon; b MEDICA Research Investigation, Hadath, Lebanon; c INSPECT-LB (Institut National de Santé Publique Epidémiologie Clinique et Toxicologie-Liban), Beirut, Lebanon; d Neuroscience Research Center, Faculty of Medical Sciences, Lebanese University, Beirut, Lebanon

**Keywords:** continuous IONM (C-IONM), intraoperative neuromonitoring (IONM), recurrent laryngeal nerve (RLN) injury, robotic thyroidectomy (RoT), surgical outcomes, vocal cord paralysis (VCP)

## Abstract

**Background:**

Intraoperative neuromonitoring (IONM) is an emerging alternative to visual identification of the recurrent laryngeal nerve (RLN) during surgery. Its goal is to reduce RLN injury and vocal cord paralysis (VCP). However, evidence has been inconsistent concerning its benefits. This study aims to assess IONM’s efficacy in preventing RLN injury during robotic thyroidectomy (RoT).

**Methods:**

This systematic review followed the Preferred Reporting Items for Systematic Reviews and Meta-Analyses (PRISMA) guidelines. A comprehensive literature search was conducted across four electronic databases: PubMed, Web of Science, EMBASE, and Cochrane Library. The inclusion criteria comprised studies published in English that focused on RoT procedures utilizing IONM to assess their effect on RLN injury. Exclusion criteria included non-English publications, studies without full-text availability, reviews, editorials, case reports, animal studies, and studies that did not meet our objectives. After screening, data were extracted and presented qualitatively.

**Results:**

An analysis of six studies involving 1006 patients, where the majority evaluated IONM within RoT procedures. Continuous IONM (C-IONM) was feasible and effective for identifying RLN and aiding in its preservation. Outcomes such as success rates, electromyography signal quality, VCP incidence, hypoparathyroidism, hypocalcemia, and bleeding showed comparable trends across groups. Notably, a study found that RoT using IONM, particularly C-IONM, was associated with significantly longer operative times averaging approximately 55.8 minutes longer than open thyroidectomy. Despite this, findings emphasized the protective role of C-IONM in minimizing nerve injury during robotic thyroid procedures, although it did not significantly impact postoperative complications like VCP or length of stay.

**Conclusion:**

Our findings suggest that IONM, particularly C-IONM, is feasible during RoT and may support RLN identification and preservation. However, current evidence is limited, and further well-designed studies are needed to clarify its clinical impact and establish criteria for routine use.

## Introduction

Robotic and endoscopic thyroidectomy have been growing rapidly globally in recent years. These minimally invasive thyroidectomy procedures have great advantages over the conventional thyroidectomy, as it has better cosmetic and functional voice outcomes^[1]^. However, damage to the RLN is one of the most serious and common complications resulting from thyroid surgery, as the incidence of temporary RLN palsy after thyroidectomy varies from 1% to 30%, while that of permanent palsy ranges from 0.5% to 5.0%^[2]^. In thyroid surgery, it is crucial to preserve the structure and function of the RLN. However, the visual identification of the RLN during thyroidectomy can be difficult because it looks like connective tissue^[3]^. Besides, the anatomic variations of the RLN, which are seen in 20% of patients, make it more susceptible to intraoperative injury^[3]^.

Intraoperative neuromonitoring (IONM) serves as a valuable supplementary tool for locating the recurrent laryngeal nerve (RLN) during thyroid surgery^[4]^. Various IONM modalities exist, including intermittent IONM (I-IONM), continuous IONM (C-IONM), and its percutaneous variant, Percu C-IONM^[5]^. IONM aids in the intraoperative detection, safeguarding, and observation of RLN function, which reduces the chances of RLN injuries and enhances vocal outcomes after surgery^[4]^. IONM provides information to surgeons about the functional integrity of the RLN, which can assist in intraoperative decisions regarding the extent of surgery, type of procedure, and airway management, particularly when bilateral RLNs are at risk. The surgeon may be able to recover damage from clipping or suturing and prevent permanent RLN injury by identifying the injured point of the RLN through serial nerve stimulation in a distal-to-proximal direction^[6]^. Furthermore, IONM may aid in recognizing the external branch of the superior laryngeal nerve responsible for voice pitch and preventing its damage, which holds particular significance for individuals who use their voice professionally^[6]^.

IONM of the RLN during thyroidectomy assists in identifying the nerve beyond direct visual methods and confirming its functional integrity, and its use is increasing globally^[7]^. However, its practicality and effectiveness in remote-access robotic and endoscopic thyroidectomy remain uncertain, as most research has focused on conventional approaches, with few studies addressing IONM specifically in robotic procedures^[7]^. Furthermore, existing studies often conflate surgical approach (robotic vs. open) with IONM use, making it difficult to isolate the effect of IONM alone. This systematic review therefore aims to evaluate the feasibility and efficacy of IONM within robotic thyroidectomy (RoT), while acknowledging that the current evidence is largely observational and does not allow definitive attribution of RLN outcomes to IONM.

## Methods

A systematic review proposal was registered with the International Prospective Register of Systematic Reviews (PROSPERO, registration number CRD42025646031). This study was designed, conducted, and reported according to the Preferred Reporting Items for Systematic Reviews and Meta-Analyses (PRISMA) Statement^[8]^. As this is a systematic review, there was no need for Ethics Committee approval and informed consent was not applicable. This manuscript complies with the TITAN Guidelines 2025 for transparency and disclosure of AI use in research and publishing^[9]^.

The review focuses on the role of neuromonitoring in preventing RLN injury (RLNI) during RoT procedures. In an effort to frame the research question, we used the PICO [population (P), intervention (I), comparator (C), and outcome (O)] framework as follows: In patients undergoing RoT (*P*), does the use of IONM (*I*) compared to RoT without neuromonitoring (*C*) reduce the incidence of RLNI (*O*)?

### Search strategy and databases

In February 2025, a comprehensive electronic search was conducted across PubMed, Web of Science, Embase, Scopus, Google Scholar, and Cochrane Library, with no restriction on publication date^[10]^. The search strategy was strategically organized using a set of MESH terms adapted for use in other bibliographic databases related to neuromonitoring, robotic surgical procedures, thyroidectomy, and RLNIs (see Supplemental Digital Content 1, available at: http://links.lww.com/MS9/B89 for the complete search strategy).

**HIGHLIGHTS**

- Continuous intraoperative neuromonitoring (IONM) is more beneficial than intermittent IONM in robotic thyroidectomy.
- IONM is valuable in high-risk patients and anatomical variations.
- Visual identification of the recurrent laryngeal nerve remains the key method.

Two independent reviewers with blind review conducted the title and abstract screening (F.Y. and A.C.), followed by the full-text screening (done independently by A.C. and A.A.B.) of the identified studies using the Rayyan platform^[11]^. Studies meeting the inclusion criteria for this systematic review were identified, and any disagreements in eligibility were resolved through consensus.

### Study selection and eligibility criteria

Our review included 1558 articles from all comparative/observational studies (including case–control, cohort, and cross-sectional studies), as well as randomized controlled trials (RCTs) and clinical trials that provided quantitative measures adequate for our review.

Included studies were confined to the English language and concentrated on patients undergoing RoT, regardless of the underlying thyroid condition (e.g., benign or malignant thyroid diseases); IONM including the use of electrophysiological monitoring during surgery to assess the functional status of the RLN in real time; and studies that specifically examined the use of neuromonitoring [e.g., electromyography (EMG) monitoring and continuous nerve monitoring] during RoT.

Excluded studies were those that focused on non-RoT techniques, including traditional open thyroidectomy, laparoscopic thyroidectomy, endoscopic thyroidectomy, or other methods not involving robotic assistance. Studies were also excluded if they lacked the use of IONM during RoT or if they compared RoT with neuromonitoring to procedures that did not involve any form of neuromonitoring. Additional exclusions included animal studies, studies involving non-human models, and those conducted in non-adult populations (under 18 years of age). Moreover, non-empirical study designs – such as study protocols, conference abstracts without full texts, and review articles – were excluded, as they do not provide original data relevant to our research question. Studies involving participants with pre-existing neurological disorders were also excluded from this review.

## Data extraction

Data extraction was carried out independently following full-text screening by the same reviewers (F.Y., A.C., and A.A.B.). To enable comparison and analysis across studies, the characteristics of each study were summed up and tabulated. Extracted information encompassed the following: basic study information (last author’s name and publication year), study design, fundamental sample characteristics (total sample size for each studied group separately, sex, and age), intervention details (type of neuromonitoring, technology used, and neuromonitoring protocol), and outcomes/results, including specific outcomes like laryngeal nerve/RLN. Only a systematic synthesis was done because the statistical combination of studies was not possible due to the variation in study components (Supplemental Digital Content 2, available at: http://links.lww.com/MS9/B90).

### Methodological quality assessment

The methodological quality assessment of the included observational studies was performed using the Newcastle–Ottawa scale (NOS), a tool designed to evaluate the quality of nonrandomized studies^[12]^. This scale examines studies based on three core domains: selection and comparability of study groups, outcome assessment for cohort and cross-sectional studies, and the quality of the data presented.

Under this framework, cohort studies can be awarded on the selection of study groups (maximum of four stars), comparability of the groups (maximum of two stars), and assessment of outcome (maximum of three stars). The total maximum score is nine stars. A study with scores from 7 to 9 has high quality, 4–6, high risk, and 0–3 present a very high risk of bias.

Comparability between groups was primarily based on age and education on the specification of the type of the surgical procedures used (endoscopic versus robotic), and technique used for nerve monitoring. Additionally, for cohort studies, a follow-up period of 12 weeks or more was considered adequate to observe outcomes.

Regarding the Joanna Briggs Institute (JBI) critical appraisal checklist, it consisted of 10 questions that assess several aspects of case series, mainly the bias risk including selection, information, and confounding bias, in addition to other factors such as inclusion/exclusion criteria and reporting. The scoring of the results is advised against since the questions are not related and they may measure different aspects; hence, the interpretation is done better by reviewing each item and the overall picture of the study^[13]^.

Two reviewers assessed them, and any concern was discussed with the team where everyone reviewed them and agreed on the results.

## Results:

### Study selection

**Figure 1.**
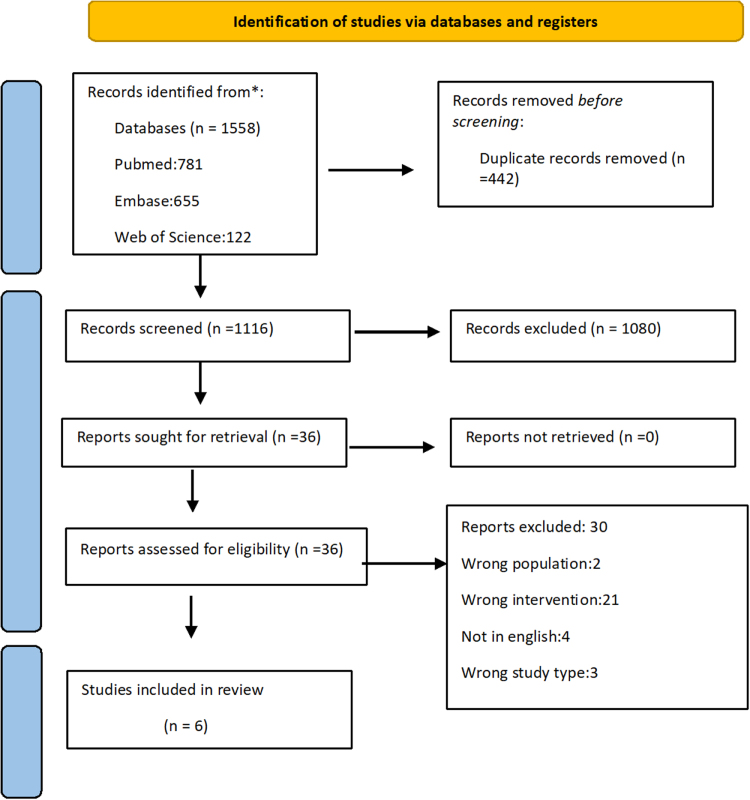
PRISMA flow chart of study selection for the systematic review on neuromonitoring in robotic thyroidectomy and its effectiveness in reducing recurrent laryngeal nerve injury.

Through database searching, the systematic review process found 1558 articles. Title and abstracts of 1116 records were examined after 442 duplicates were eliminated; 36 were chosen for full-text review. The PRISMA flow chart is illustrated in Figure 1, which shows the sequence of the study selection; six of these studies met the criteria and focused on IONM in RoT versus conventional techniques.

### Study characteristics

The six studies were done in Korea (*n* = 5) and China (*n* = 1). Patient counts ranged from 30 to 304 in the 1213 total samples (mean age 42–58 years). Among the surgical techniques used were robotic dual or bilateral axillo-breast approach (BABA), retroauricular, and transoral thyroidectomy. Pathologies ranged from benign nodules to papillary thyroid carcinoma^[7]^. All the studies’ characteristics are implicated in Supplemental Digital Content Table S1, available at: http://links.lww.com/MS9/B88.

### Risk of bias and quality assessment

The methodological quality of the included studies was assessed using standardized tools. Cohort and case–control studies were evaluated using the NOS, while the single case series was assessed using the JBI critical appraisal checklist for case series.

Based on the NOS shown in Table 1, the overall methodological quality of the included studies was high, with five studies scoring 8 out of 9 and one study scoring 6 out of 9^[1]^. This suggests a low overall risk of bias, although the moderate-quality study introduces some uncertainty.

**Table 1:** Quality assessment of included cohort studies using the Newcastle–Ottawa scale

ID	Study	Study design	Selection	Comparability	Outcome	Score
1	Paek *et al*. (2022)^[13]^	Retrospective cohort	★★★★	★★	★★★	8
2	Ji *et al*. (2020)^[7]^	Retrospective cohort	★★★★	★★	★★★	8
3	Lee *et al*. (2024)^[1]^	Retrospective case–control	★★★★	★★	★★★	6
4	Zhang *et al*. (2022)^[5]^	Prospective cohort	★★★★	★★	★★★	8
5	Bae *et al*. (2015)^[3]^	Retrospective cohort	★★★★	★★	★★★	8

The case series study^[14]^ employed the measurement of the RLN condition using a standardized method for all participants, as shown in Table 2, from the same equipment (EMG NIM 3.0 system) and measurements (regular checks of electrode impedance). However, the detection of the laryngeal nerve twitch was different, where endoscopic visualization and magnification were used in the RAT versus direct palpation in COT.

**Table 2:** JBI critical appraisal checklist for the case series done by Ban *et al*

Criteria	Answers
1. Were there clear criteria for inclusion in the case series?	Unclear
2. Was the condition measured in a standard, reliable way for all participants included in the case series?	Yes
3. Were valid methods used for identification of the condition for all participants included in the case series?	Yes
4. Did the case series have consecutive inclusion of participants?	Unclear
5. Did the case series have complete inclusion of participants?	Unclear
6. Was there clear reporting of the demographics of the participants in the study?	Yes
7. Was there clear reporting of clinical information of the participants?	Yes
8. Were the outcomes or follow-up results of cases clearly reported?	Yes
9. Was there clear reporting of the presenting site(s)/clinic(s) demographic information?	No
10. Was statistical analysis appropriate?	Yes

Neuromonitoring techniques included I-IONM, C-IONM, and percutaneous C-IONM. Devices included the NIM 3.0 System (Medtronic) and EMG endotracheal tubes^[3,5]^.

### Comparators

Four studies compared RoT with IONM to COT^[1,7,14,15]^. Neuromonitoring techniques were directly compared in two studies: percutaneous C-IONM (100% feasibility, 304/304) vs. I-IONM (82.7% success rate, 86/104)^[5,7]^. Surgical techniques included BABA (*n* = 3), retroauricular (*n* = 2), and transoral (*n* = 2)^[5,7]^ .

### Intervention application

Surgical duration for robot surgery spanned from 112.8 to 204.8 minutes^[14,15]^. Surgeons (*n* = 5) or expert technicians (*n* = 2) conducted IONM using vagal nerve stimulation, RLN mapping, and EMG signal analysis.

### Feasibility and practicality of IONM in RoT

C-IONM showed high feasibility and practical utility in RoT^[5]^. C-IONM detected electromyographic (EMG) amplitude reductions in 48/323 RLNs, allowing for timely intraoperative intervention to prevent irreversible nerve injury. Percutaneous C-IONM achieved 100% feasibility in 304 patients^[5]^, while remote-access RoT showed 82.7% feasibility in 86/104 patients^[7]^. I-IONM, in contrast, failed to detect EMG abnormalities in 0/100 RLNs. Across robotic procedures, RLN identification was consistently reliable, with all 56 nerves visualized under IONM guidance^[3]^.

### Effectiveness in nerve preservation and clinical outcomes

C-IONM contributed to nerve preservation by enabling real-time modifications during dissection, lowering irreversible RLN injury rates (48/323 RLNs). Clinical outcomes in RoT included:

1. Transient vocal cord paralysis (VCP): 6.0%^[1]^.
2. Permanent VCP: 0%^[14]^.
3. Transient hypoparathyroidism: 1.9%^[15]^.
4. Hypocalcemia: 2/304 patients (percutaneous C-IONM).
5. No postoperative bleeding was reported^[1,3]^.
6. EMG signal amplitudes >500 µV were observed in 57% of RLNs in remote-access procedures^[7]^.

**Figure 2.**
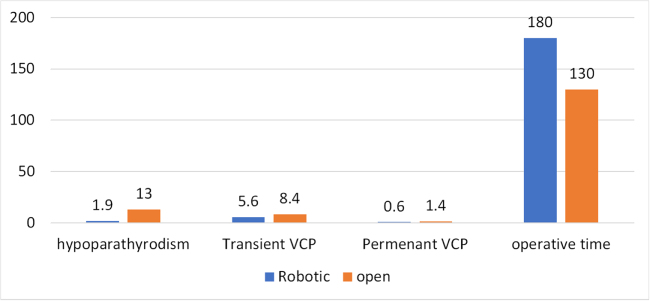
Comparing different outcomes between robotic and open thyroidectomy.

Figure 2 illustrates differences in outcomes between robotic and open thyroidectomy.

### Comparisons across IONM modalities and surgical approaches

- C-IONM vs. I-IONM: C-IONM detected EMG amplitude reductions in 48/323 RLNs, whereas I-IONM detected none (0/100 RLNs), showing higher sensitivity for intraoperative nerve monitoring^[5]^.
- Robotic vs. open thyroidectomy: Robotic procedures with IONM had lower rates of transient hypoparathyroidism (1.9%)^[15]^ and no permanent VCP. Transient VCP rates were similar across approaches (6.0%), and the median length of stay was comparable (3 days).

**Figure 3.**
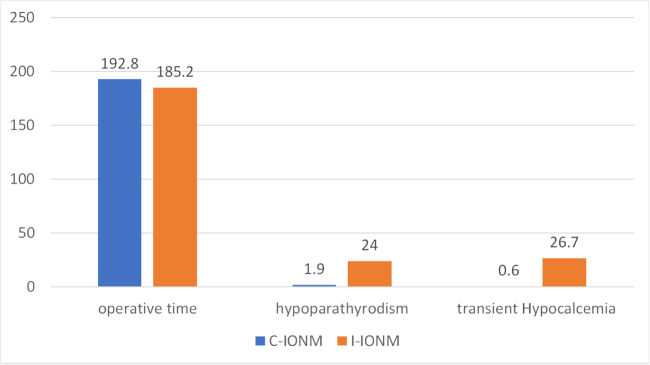
Comparing different outcomes between C-IONM and I-IONM.

Figure 3 illustrates some different outcomes assessed comparing C-IONM to I-IONM.

### Operative time

RoT using the BABA with C-IONM was related to longer operative durations: 192.8 minutes^[15]^. Likewise, transoral RoT employing I-IONM needed 185.2 minutes^[3]^.

### Postoperative complications

The incidence of transient VCP showed no significant difference between robotic and open thyroidectomy groups, occurring in 6.0% of patients in both cohorts^[1]^. However, permanent VCP was absent in robotic procedures (0%)^[14]^. Length of stay was comparable across approaches, with a median of 3 days for robotic groups^[5]^. C-IONM demonstrated a protective role, preventing permanent RLN injury in 10/304 patients who experienced transient VCP^[5]^.

## Discussion

This systematic review aimed to evaluate the effectiveness of IONM in reducing the risk of RLN injury during RoT. Our findings suggest that IONM, particularly C-IONM, may support RLN identification and intraoperative detection of nerve abnormalities. However, it remains difficult to attribute improved outcomes solely to IONM. Most included studies compared RoT with IONM to open thyroidectomy without IONM, introducing confounding between the surgical approach and the neuromonitoring technique. The apparent benefits observed in IONM-assisted robotic procedures may therefore reflect procedural characteristics of robotic surgery, surgeon *proficiency*, or *institutional* expertise rather than the independent effect of IONM. These observations are consistent with the 2023 meta-analysis by Cozzi *et al*, which pooled data from over 73 000 nerves and found no significant reduction in temporary or definitive RLN injury with IONM, and largely overlapping injury rates between continuous and intermittent IONM^[16]^. This evidence underscores the need for cautious interpretation of IONM’s protective role and highlights the importance of future well-designed, multicenter randomized trials to determine its true clinical impact in RoT.

Three surgical techniques were used in the retrieved studies regarding RoT: BABA, transoral (TORT), and retroauricular (facelift). BABA uses two incisions in the breast (circumareolar region) and the axillary fossa, allowing a view that resembles that of traditional surgery. It is excellent for total thyroidectomies^[17]^. On the other hand, TORT can include sublingual and vestibular approaches. Due to the short distance from such areas to the thyroid gland, this technique requires less tissue dissection and allows total thyroidectomy^[17]^. For the facelift approach, incisions behind the ear and in the occipital hairline are created. What makes it less favorable than the previous two approaches is the difficulty of performing a contralateral lobectomy through a unilateral incision. So, it is preferred just for thyroid lobectomies^[17]^.

C-IONM showed high feasibility, with successful application in 82.7% of remote-access RoT cases^[7]^ and 100% in percutaneous C-IONM^[5]^. Our findings are in line with the results drawn by Dionigi *et al*^[18]^ that ensure the feasibility of IONM since no malfunctions were documented. Also, IONM was applied in 75% of the studies during all robotic and endoscopic surgeries^[19]^.

Transient VCP was the same between robotic and open surgery groups (6.0% each), while permanent VCP was absent in the robotic IONM group compared to a small but notable incidence (1.4%) in the open surgery^[14]^. Although the difference may not reach statistical significance due to limited sample sizes, it hints at a protective role of IONM in preserving long-term vocal cord function, and this will reflect the postoperative dysphagia, hoarseness, and voice quality, so this type of surgery may be more relevant for patients whose professions rely on vocal performance. Despite promising trends, current evidence does not consistently demonstrate statistically significant reductions in RLN injury with IONM compared to visual identification alone, particularly in low-risk cases. This aligns with previous meta-analyses showing mixed results on its effectiveness^[20]^.

Furthermore, complications such as hypoparathyroidism and hypocalcemia were less frequent in robotic surgeries with IONM. Transient hypoparathyroidism was significantly lower in the robotic group (1.9%) compared to the open group (13.0%)^[15]^, and only two patients having percutaneous C-IONM had hypocalcemia^[5]^, suggesting that the combination of robotic access and IONM may contribute to overall gland preservation and reduced tissue trauma.

Although no significant differences were found in hospital length of stay or bleeding, the robotic procedures were consistently associated with longer operative times averaging up to 55.8 minutes more than conventional open surgeries^[14,15]^. This shows that robotic surgery is more time-consuming, influenced by the robotic setup, and may have more complications related to operation duration

While IONM, particularly C-IONM, does not completely eliminate the risk of RLN injury, its application in RoT appears to enhance surgical precision and safety. The ability to identify and monitor the functional integrity of the nerve in real-time provides a strategic advantage in preventing complications such as VCP, dysphagia, and hoarseness.

### Implications

The findings of this systematic review carry important clinical, theoretical, and practical implications for the evolution of thyroid surgery, particularly in the use of robotic techniques.

This review highlights the importance of using IONM, particularly continuous C-IONM, to improve patient safety during RoT. The incidence of RLN injury is low in both robotic and conventional surgeries. However, C-IONM’s capability to provide real-time feedback during dissection allows for prompt intraoperative intervention. This proves especially advantageous in situations that involve complex anatomy or are high-risk, as even minor nerve damage can result in considerable postoperative morbidity. The lower rates of permanent VCP and transient hypoparathyroidism further indicate that IONM can help enhance patients’ functional outcomes and quality of life.

C-IONM represents a shift toward integrating neurophysiological monitoring into surgical decision-making and moving beyond the traditional reliance on visual identification alone^[19]^. This can be a way for new standardized nerve preservation protocols, especially in minimally invasive and robotic-assisted approaches.

Moreover, the adoption of IONM despite the longer operative time can be seen as a strategic investment in surgical precision and patient-centered care. As RoT becomes more common, especially in regions prioritizing cosmetic outcomes and minimally invasive access, the integration of IONM could become a best practice, particularly in centers of excellence or high-volume institutions^[21]^. Furthermore, the widespread use of IONM may help in training and skill development for surgical residents and fellows by providing real-time feedback on nerve handling and technique^[22]^.

### Limitations

Our review has several limitations. Most included cohorts had relatively small sample sizes, limiting generalizability and statistical power. All studies were observational, resulting in moderate certainty of evidence and preventing definitive attribution of RLN outcomes to IONM. Furthermore, variability in study designs, patient populations, and surgical expertise may have influenced reported outcomes. A key limitation is the inability to isolate the effect of IONM from that of the surgical approach, as most studies compared RoT with IONM to open thyroidectomy without IONM. Consequently, the specific protective role of IONM cannot be conclusively established. The absence of well-designed RCTs further weakens the strength of the evidence. Therefore, larger multicenter RCTs are needed to confirm these findings and support the standardized implementation of IONM in RoT.

Furthermore, heterogeneity was noted in surgical approaches (e.g., BABA, retroauricular, and transoral), types of IONM used (intermittent vs. continuous and percutaneous vs. standard), and EMG interpretation protocols, making direct cross-study comparisons challenging. Key outcomes such as postoperative voice quality, hoarseness, and dysphagia were also inconsistently reported across studies. Finally, this review included only English-language publications and excluded unpublished studies, raising the possibility of language and publication bias.

## Conclusion

RLN injury remains a significant complication of thyroidectomy, potentially leading to temporary or permanent VCP and affecting patients’ quality of life. Thyroidectomy continues to be a major cause of RLN injury, highlighting the need for strategies to improve nerve preservation. Our review suggests that IONM, including C-IONM, is feasible during RoT and may support RLN identification and detection of intraoperative nerve abnormalities. However, direct evidence demonstrating a definitive reduction in postoperative nerve injury or VCP remains limited, and observed benefits may reflect intraoperative signal changes rather than patient-important outcomes. Comparisons between C-IONM and I-IONM are largely descriptive, with differences in feasibility and EMG amplitude reduction noted, but no statistically pooled analysis of critical outcomes such as VCP rates is available. Therefore, the current evidence does not allow firm conclusions regarding the superiority of C-IONM over I-IONM. To confirm the potential clinical benefits of IONM in RoT, large multicenter RCTs are needed that compare IONM to visual identification alone and assess patient-centered outcomes, including RLN injury and vocal cord function.

## Data Availability

All data generated or analyzed during this study are included in this published article and its supplementary materials. No additional datasets were generated or analyzed.

## Research registration unique identification number (UIN)

PROSPERO, registration number CRD42025646031.

## Presentation

Poster presentation at the 5th Lebanese University Medical Assembly; accepted for presentation at the minimally invasive surgery conference in Florida, USA.

## References

[1] Lee JH Kwon H. An institutional experience of intraoperative neurophysiological monitoring application in robotic thyroidectomy: a retrospective case-control study. Ann Surg Treat Res 2024;106:243–47. 38725805 10.4174/astr.2024.106.5.243 PMC11076952

[2] Lukinović J Bilić M. Overview of thyroid surgery complications. Acta Clin Croat 2020;59:81–86. 10.20471/acc.2020.59.s1.10 PMC8212606 34219888

[3] Bae DS Jin KS. Intraoperative neuromonitoring of the recurrent laryngeal nerve in robotic thyroid surgery. Surg Laparosc Endosc Percutan Tech 2015;25:23. 25238177 10.1097/SLE.0000000000000074

[4] Group with the IIMS, Randolph GW Dralle H. Electrophysiologic recurrent laryngeal nerve monitoring during thyroid and parathyroid surgery: international standards guideline statement. Laryngoscope 2011;121:S1–S16. 21181860 10.1002/lary.21119

[5] Zhang D Wang C Wang T. Clinical experience of use of percutaneous continuous nervemonitoring in robotic bilateral axillo-breast thyroid surgery. Front Endocrinol 2022;12:817026. 10.3389/fendo.2021.817026 PMC8862684 35211092

[6] Choi SY Son YI. Intraoperative neuromonitoring for thyroid surgery: the proven benefits and limitations. Clin Exp Otorhinolaryngol 2019;12:335–36. 31575106 10.21053/ceo.2019.00542 PMC6787475

[7] Ji YB Ko SH Song CM. Feasibility and efficacy of intraoperative neural monitoring in remote access robotic and endoscopic thyroidectomy. Oral Oncol 2020;103:104617. 32126516 10.1016/j.oraloncology.2020.104617

[8] Page MJ McKenzie JE Bossuyt PM. The PRISMA 2020 statement: an updated guideline for reporting systematic reviews. Published online March 29, 2021. doi:10.1136/bmj.n71 PMC8008539 33781348

[9] Transparency In The reporting of Artificial INtelligence – the TITAN guideline - Premier Science. Accessed September 15, 2025. https://premierscience.com/pjs-25-950/

[10] Bramer WM Rethlefsen ML Kleijnen J. Optimal database combinations for literature searches in systematic reviews: a prospective exploratory study. Syst Rev 2017;6:245. 29208034 10.1186/s13643-017-0644-y PMC5718002

[11] Ouzzani M Hammady H Fedorowicz Z. Rayyan—a web and mobile app for systematic reviews. Syst Rev 2016;5:210. 27919275 10.1186/s13643-016-0384-4 PMC5139140

[12] Bae JM. A suggestion for quality assessment in systematic reviews of observational studies in nutritional epidemiology. Epidemiol Health 2016;38:e2016014. 27156344 10.4178/epih.e2016014 PMC4877518

[13] Munn Z Barker TH Moola S. Methodological quality of case series studies: an introduction to the JBI critical appraisal tool. JBI Evid Synth 2020;18:2127. 33038125 10.11124/JBISRIR-D-19-00099

[14] Ban MJ Chang EHE Lee DY. Analysis of neuromonitoring signal loss during retroauricular versus conventional thyroidectomy. Laryngoscope 2019;129:2199–204. 30585327 10.1002/lary.27749

[15] Paek SH Kwon H Kang KH. A comparison of the bilateral axillo-breast approach (BABA) robotic and open thyroidectomy for papillary thyroid cancer after propensity score matching. Surg Laparosc Endosc Percutan Tech. Published online 2022 doi:10.1097/SLE.0000000000001085 36044331

[16] Cozzi AT Ottavi A Lozza P. Intraoperative neuromonitoring does not reduce the risk of temporary and definitive recurrent laryngeal nerve damage during thyroid surgery: a systematic review and meta-analysis of endoscopic findings from 73,325 nerves at risk. J Pers Med 2023;13:1429. 37888040 10.3390/jpm13101429 PMC10607766

[17] Tae K. Robotic thyroid surgery. Auris Nasus Larynx 2021;48:331–38. 32636045 10.1016/j.anl.2020.06.007

[18] Dionigi G Kim HY Wu CW. Neuromonitoring in endoscopic and robotic thyroidectomy. Updat Surg 2017;69:171–79. 10.1007/s13304-017-0442-z 28439772

[19] Barczyński M Randolph GW Cernea CR. External branch of the superior laryngeal nerve monitoring during thyroid and parathyroid surgery: International Neural Monitoring Study Group standards guideline statement. Laryngoscope 2013;123:S1–S14. 10.1002/lary.24301 23832799

[20] Hotouras A Murphy J Abeles A. Symptom distribution and anorectal physiology results in male patients with rectal intussusception and prolapse. J Surg Res 2014;188:298–302. 24411299 10.1016/j.jss.2013.12.008

[21] Boonstra PA van Veen RN Stockmann HBAC. Less negative appendectomies due to imaging in patients with suspected appendicitis. Surg Endosc 2015;29:2365–70. 25475515 10.1007/s00464-014-3963-2

[22] Dralle H Sekulla C Lorenz K. German IONM Study Group. Intraoperative monitoring of the recurrent laryngeal nerve in thyroid surgery. World J Surg 2008;32:1358–66. 18305996 10.1007/s00268-008-9483-2

